# Neurophysiological Hallmarks of Neurodegenerative Cognitive Decline: The Study of Brain Connectivity as a Biomarker of Early Dementia

**DOI:** 10.3390/jpm10020034

**Published:** 2020-04-30

**Authors:** Paolo Maria Rossini, Francesca Miraglia, Francesca Alù, Maria Cotelli, Florinda Ferreri, Riccardo Di Iorio, Francesco Iodice, Fabrizio Vecchio

**Affiliations:** 1 Brain Connectivity Laboratory, Department of Neuroscience & Neurorehabilitation, IRCCS San Raffaele Pisana, 00167 Rome, Italy; fra.miraglia@gmail.com (F.M.); francesca.alu@sanraffaele.it (F.A.); franc.iodice@gmail.com (F.I.); fabrizio.vecchio@uniroma1.it (F.V.); 2 Neuropsychology Unit, IRCCS Istituto Centro San Giovanni di DioFatebenefratelli, 25125 Brescia, Italy; mcotelli@fatebenefratelli.eu; 3 Department of Neuroscience, Unit of Neurology and Neurophysiology, University of Padua, 35100 Padua, Italy; fimferreri@yahoo.it; 4 Department of Clinical Neurophysiology, Kuopio University Hospital, University of Eastern Finland, 70100 Kuopio, Finland; 5 Neurology Unit, IRCCS Polyclinic A. Gemelli Foundation, 00168 Rome, Italy; riccardo.diiorio@policlinicogemelli.it

**Keywords:** Alzheimer’s disease, mild cognitive impairment, EEG, TMS

## Abstract

Neurodegenerative processes of various types of dementia start years before symptoms, but the presence of a “neural reserve”, which continuously feeds and supports neuroplastic mechanisms, helps the aging brain to preserve most of its functions within the “normality” frame. Mild cognitive impairment (MCI) is an intermediate stage between dementia and normal brain aging. About 50% of MCI subjects are already in a stage that is prodromal-to-dementia and during the following 3 to 5 years will develop clinically evident symptoms, while the other 50% remains at MCI or returns to normal. If the risk factors favoring degenerative mechanisms are modified during early stages (i.e., in the prodromal), the degenerative process and the loss of abilities in daily living activities will be delayed. It is therefore extremely important to have biomarkers able to identify—in association with neuropsychological tests—prodromal-to-dementia MCI subjects as early as possible. MCI is a large (i.e., several million in EU) and substantially healthy population; therefore, biomarkers should be financially affordable, largely available and non-invasive, but still accurate in their diagnostic prediction. Neurodegeneration initially affects synaptic transmission and brain connectivity; methods exploring them would represent a 1st line screening. Neurophysiological techniques able to evaluate mechanisms of synaptic function and brain connectivity are attracting general interest and are described here. Results are quite encouraging and suggest that by the application of artificial intelligence (i.e., learning-machine), neurophysiological techniques represent valid biomarkers for screening campaigns of the MCI population.

## 1. Introduction

Dementias are of several types; however, the most frequent and diffusely known by the public opinion is Alzheimer’s disease (AD), which is characterized by a progressive loss of memory and deterioration of other cognitive functions that significantly interfere with daily life activities [1]. The typical AD clinical phenotype follows a prodromal stage known as mild cognitive impairment (MCI) that is usually, but not exclusively, characterized by memory loss (amnestic MCI = aMCI). MCI is typically characterized by evidence of an objective impairment of memory and/or of other cognitive domains on neuropsychological testing, but not yet encompassing the standards for dementia diagnosis. It represents an intermediate condition in the elderly between normal cognition and dementia and includes a consistent percentage of subjects (about 50%) in a stage that is prodromal to different types of dementia, including AD (MCI prodromal-to-dementia or prodromal-to-AD). MCI is considered a high-risk population since a significant percentage (from 5 to 20 times higher compared to an age-matched non-MCI population) will develop one type of dementia during a 3- to 5-year follow-up period; the remaining percentage will stay in the MCI condition for the rest of their life or even revert to full normality. MCI prodromal-to-AD (or due-to-AD) cannot be distinguished from those who will not convert on purely clinical grounds. A careful MCI definition requires a comprehensive assessment, including cognitive complaints questionnaires, screening tests (such as Mini-Mental State Examination (MMSE)), an in-depth neuropsychological evaluation (including tests for episodic memory, language, visuo–spatial abilities, and behavioral scales with appropriate normative thresholds [2,3]), functional scales and full neurological examinations. In order to plan optimal and early therapeutic, organizational, and rehabilitative interventions, MCI diagnosis should be combined with the most reliable prognosis on the likelihood and time of eventual progression to dementia. In other words, those MCI subjects who are already in a prodromal-to-dementia condition should be intercepted as early as possible. This goal can nowadays be achieved by combining biomarkers reflecting ongoing neurodegenerative phenomena with the results of neuropsychological tests.

The identification of reliable markers able to intercept those MCI subjects (amnesic, non-amnesic, multi-domain) who are in a prodromal-to-dementia stage represents a goal for all health systems as it would allow early interventions on different risk factors. The risk factors include lifestyle aspects such as obesity, sedentary lifestyle, smoke, low daily cognitive and exercise, and medical conditions such as cardiovascular diseases, diabetes, hypercholesterolemia, and thyroid dysfunction, leading to a significant delay in the daily living autonomies loss even in the absence of a disease-modifying therapy [4,5,6,7,8,9,10]. Such a goal would be of paramount importance since—just as an example that might be equally expanded to all countries with an aging population—the costs of dementia in the United States (US) were estimated to be USD 818 billion in 2015, with an increase of 35% compared to 2010. Moreover, MCI prodromal-to-AD subjects are the main targets of many clinical trials with potentially disease-modifying drugs since these drugs have proved ineffective when full symptomatology of AD has already developed, probably because the “neural reserve” has been progressively consumed during the pre-symptomatic and prodromal disease stages. Therefore, early markers predicting with high sensitivity/specificity the evolution from prodromal stages to clinically overt dementia and AD are of pivotal importance in modern public health strategies.

Within this theoretical frame, it seems quite important to have a 1st-level type of low-cost, non-invasive, and widely available biomarker(s) able to screen out from the MCI population those subjects who are non-prodromal-to-dementia, leaving more expensive and highly demanding technologies as a 2nd level approach for a significantly smaller population with a remarkably higher risk of being in a prodromal-to-dementia condition for diagnostic characterization (i.e., AD with amyloid plaques).

## 2. EEG Biomarkers

Scalp resting state electroencephalographic (EEG) rhythms reflect the summation of oscillatory membrane post-synaptic potentials generated from cortical pyramidal neurons, which play the role of electromagnetic signal sources. These sources were estimated to extend for several square centimeters of the brain cortex [11,12]. These potentials can be considered as the oscillatory output of the resting state cortical system, while inputs include afferents coming from other cortical neural biomasses, thalamo-cortical neurons, and neurons belonging to ascending reticular systems [11].

Practically speaking, EEG data analysis may be divided into a two-step process: first, the signals recorded from all sensors are “de-noised”, aiming to improve signal-to-noise ratio by excluding portions of highly noisy data; second, the current density distribution or other parameters of interest are estimated from the cleaned sensor recorded signals. This phase, called preprocessing, is devoted to the extraction of the source under study from the whole population of electromagnetic sources, including also the artefactual ones. Methods improving the signal/noise ratio “separate as much as possible the signal from the noise using information on the specific source under study. In some cases, it is possible to observe neural activity synchronization by supplying to the subject an external stimulus or instructing the subject to perform a specific task. Given the high relevance of analyzing resting-state activity, alternative procedures to enhance the signal to noise ratio were developed, including Blind Source Separation (BSS) methods such as Independent Component Analysis—ICA [13] and semi-BSS methods such as Functional Source Separation—FSS” (see Figure 2) [14,15].

Another important step aims to determine the current density distribution inside the brain, especially in the region of interest. The diverse approaches to solve the so-called inverse-problem (that is the identification of the source(s) within the brain responsible for the distribution of scalp-recorded electromagnetic signals) range from single and multiple dipoles [16] to distributed sources, which include the Multiple Signal Classification—MUSIC [17], the recursively applied and projected MUSIC—RAP-MUSIC [18], the minimum norm estimates—MNE [19], the low-resolution brain electromagnetic tomography—LORETA [20], and the beam-forming and synthetic aperture magnetometry—SAM [21].

The scalp-recorded EEG signals oscillate with rhythms characterized by a spectral content below 50 Hz since the extracerebral layers act as spatial and frequency filters. Two classes of EEG biomarkers for early dementia diagnosis, such as “synchronization” and “connectivity” can be nowadays identified [22]. The term “synchronization” refers to nonlinear oscillatory components of the brain system that reflect a collective oscillatory behavior of cortical neural populations generating EEG rhythms [23]. Synchronization of the cyclic firing of cortical neural populations is the main source of scalp EEG rhythms in both resting state and task-related conditions and produce scalp EEG rhythms: this “synchronization” mechanism must occur at a macroscopic spatial scale of some centimeters. Spectral analysis of EEG rhythms is typically done at fixed frequency bands. Both nonlinear and linear mathematics can estimate the neural current density of EEG cortical sources [24,25]. These procedures model 3D tomographic patterns of EEG cortical generators into a spherical or a magnetic resonance imaging (MRI)-based head model representing electrical properties of the cerebral cortex, skull, and scalp, typically co-registered to Talairach brain atlas [26,27,28,29,30]. Source localization procedures estimate the current intensity of all dipoles (e.g., hundreds to thousands) of the cortical mantle model to explain scalp EEG amplitude/power density.

### 2.1. EEG Findings in Dementia (including AD)

It is important to clarify that EEG recordings (particularly the routine ones with 19 electrodes) cannot reach a distinct diagnosis of the various types of dementia. In all the studies reported below, the diagnosis of AD was reached with neuropsychological tests eventually combined with other biomarkers dealing with brain metabolism and analysis of beta-amyloid and tau protein metabolites (i.e., fluorodeoxyglucose positron emission tomography (PET–FDG), PET–radioligands, and cerebrospinal fluid (CSF) analysis). Having clarified this important point, one should consider that there is a vast literature on EEG abnormalities in pathological brain aging (for a review, see [31]). Compared to cognitively intact elderly (Nold) subjects, demented (namely, AD) patients contain excessive δ and a significant decrement of posterior α rhythms [32]. Similarly, MCI patients display a significant decrease of α power compared to Nold [33]. Furthermore, a prominent decrease of EEG spectral coherence in the α band in AD has been reported [34,35]. Indeed, the EEG power spectrum in patients with AD compared to age-matched Nold has shown a widespread increase in δ and θ power density and a posterior decrease in α and β power density with lowering of α power density peak in several studies [34,35,36,37,38].

Nonlinear measures of “synchronization” markers pointed to a complexity loss of cerebral dynamics in AD within the same frequency bands [39,40,41,42,43,44,45], while the analysis of phase coherence showed differences between AD and Nold [35] and was also able to predict aMCI conversion to AD as demonstrated by neuropsychological follow-up [34]. Cross-validation of EEG source solutions showed that clinical symptoms were positively correlated with abnormalities in β, α, and δ source activities [46,47]. Global cognitive status, as revealed by MMSE scores, correlated negatively with δ/θ source activity and positively with α source activity [22,47,48,49]. Similar features of EEG sources with some attenuation in amplitude, as seen in AD patients, were also observed in MCI subjects [22,49]. These findings were confirmed by an independent approach based on minimum-norm depth-weighted estimation [50], that showed in AD patients a reduced activity in the precuneus, posterior cingulate, and parietal regions, as well as increased activity in δ or θ sources in inferior parietal cortex, medial temporal cortex, precuneus, and posterior cingulate, compared to aMCI subjects [50].

Occipital, temporal, and parietal α source activities correlated with hippocampal volume, being more evident in aMCI subjects with a greater volume, intermediate in those with a smaller volume, and minimum in AD patients [47]. Moreover, α source activity was statistically linked to the volume of cortical gray matter in aMCI and AD subjects, while a negative correlation was found with δ sources [51,52]. Finally, a negative correlation between EEG α dipolarity (e.g., uniformity of α potential distribution) and p-tau or p-tau/Aβ ratio in cerebrospinal fluid in AD was described [53].

Nonlinearity brain electromagnetic rhythms have attracted substantial attention since the early 1980s [42,54,55], due to the approach based on the chaos theory, aiming at a deterministic characterization of complex time series [56,57] and to the observation that multiple neural processes are governed by nonlinear phenomena which are essential for healthy and adaptive cortical activity, but are also involved in several brain diseases [58]. The early application of nonlinear methods based on the chaos theory to the analysis of spontaneous EEG in AD showed lower correlation dimension (D2) [56] and the largest Lyapunov exponent (L1) [57] compared to Nold, attributable to a reduction of the variables needed to describe the dynamics of the EEG (D2) and to a loss of flexibility in information processing (L1). This is because D2 is a measure of the geometry of the attractor that describes the EEG signals, whereas L1 explains how many similarities diverge over time [54]. Despite their different focus on static and dynamic properties of the ongoing signals, both D2 and L1 parameters paralleled the reduction of complexity seen in the EEG activity of AD patients [45,54,59,60,61].

Methods of nonlinear EEG analysis can be categorized into three main groups:

- Fractal dimension metrics, including Katz and Higuchi’s definitions [62,63].
- Irregularity estimators, including sample entropy [64] and permutation entropy [65].
- Multiscale metrics [66], including multiscale sample entropy and derived approaches such as multiscale dispersion entropy [45].

The concept of fractal dimension refers to a non-integer dimension of a geometric object; this parameter is reduced in AD compared to Nold, especially in temporal–occipital regions [61]. Metrics such as sample entropy (SampEn) can be seen as measures of the production rate of information within a signal (how much information previous samples of the time series provide about the following samples) and its level of predictability [61]. Entropy metrics of spontaneous EEGs showed reduced irregularity in AD. The third major category of nonlinear measures is related to the multiscale behavior of signals and to the concept of complexity ranging between two extremes of fully predictable and deterministic systems and merely random oscillations [67]. Thus, completely ordered (i.e., predictable) or random systems are not physiologically complex [68]. A working measure of complexity (defined as multiscale (sample) entropy (MSE [67])) is based on the measure of entropy (originally SampEn) over multiple temporal scales obtained from “coarse-grained” versions of the signals [60,66], and it has inspired the application of entropy metrics in a multiscale way [66]. MSE has been compared between AD and Nold [55,68,69]; it has been shown that spontaneous EEG activity in AD is less complex at short temporal scales (associated with higher frequencies), but this tendency reverses at longer temporal scales (related to lower frequencies) [68,69]. This finding is of remarkable interest when arguing about the dependency of the complexity of brain EEG activity on the temporal scales and the frequency range under analysis [70,71]. Arguably, one of the limitations of the nonlinear methods surveyed so far is that they are applicable to single (univariate) signals only. Multivariate versions have become recently available [45,72,73,74]; however, they should be validated more deeply as a probe for EEG analysis. Finally, we should consider that non-linear analysis of EEG activity has been explored in resting-state and awake conditions; methods also applicable to short time series can now be utilized before, during, and after a task with the aim of increasing sensitivity/specificity to characterize pathological cognitive decline [59,75,76].

Despite a number of limitations, important recent reviews ([44], Rossini et al. (2020)) have summarized the progress in the EEG pattern of demented patients with a neuropsychological profile of AD: generalized slowing of the spectral frequency profile, reduced complexity, and perturbation.

### 2.2. Brain Connectivity Methods including Graph-Theory

The human brain can be represented as an anatomic-functional matrix (consisting of billions of neurons and their synaptic connections) of network structures at micro–meso–macro-scale levels. Within this matrix of networks, nodes (neuronal assemblies) and links (connecting fibers) cooperate via dynamic aggregations or transient locking/unlocking of their orchestrated firing oscillations [77,78,79,80]. Networks continuously re-shape throughout life via plastic modifications mainly governed by long term synaptic potentiation/depression (LTP/LTD) mechanisms ruled by the continuous input bombardment from internal and external environments, including learning/training and maturation/aging processes. Network configuration and excitability are continuously changing even in tens of millisecond time frames, according to the cyclic changes of the cortical state (“cortical uncertainty” of Adrian and Moruzzi, 1939 [81]). Such continuous variability modifies instant-by-instant the efficacy of the brain networks supporting a given skill or task. On this basis, it can be explained why an operating subject can incur cyclic errors during a task even if in apparently stable conditions. Phase synchronization (or coherence), phase-locking, entrainment, cross-frequency (or power synchrony), and phase reset of EEG rhythms measure the degrees of functional and effective connectivity between different brain areas [5,82,83]. As previously said, electromagnetic brain signals are generated by neuronal activities having millisecond time constants and have, therefore, an extremely high temporal discrimination. Because of this, by examining them, one can theoretically follow the dynamics and hierarchies of neuronal assembly connection/disconnection in analogy to the binding/unbinding phenomena of neuronal firing phase coherence, as seen in animal models via microelectrodic recordings. Similar conditions have been recorded in human depth recordings where synchronization mechanisms have been observed to be highly correlated with cognitive performance [84,85].

The stationarity of the resting-state cerebral system (as opposed to non-stationarity) means that the statistical features of scalp electromagnetic brain rhythms are constant during recordings. Stationary conditions can be observed for relatively short periods, usually not longer than tens of seconds [86], during which electromagnetic rhythms can be examined by classical linear frequency analysis [87,88]. Linearity and non-linearity describe the behavior of a neural circuit, in which the output signal strength varies in direct or non-direct proportion to the input signal strength, respectively.

Several tools for EEG analysis exploit graph theory [89], which returns indicators of the balance between the local connectedness and the global integration of a network matrix. Time series of cortical electric neuronal activity can be used for estimating cortical connectivity, based on the following concept: “Two places are functionally connected if their activity time series are similar” in which the ‘two places’ could be replaced by ‘two neuronal assemblies’ or ‘the neuronal assemblies under two recording electrodes’ [90]. However, from a formal point of view, there are many different ways to define the similarity between signals, including those from EEG. Such methods are mainly based on the exact low-resolution electromagnetic tomography (eLORETA) [91], an algorithm representing a linear inverse solution for EEG signals that have no localization error to point sources under ideal (noise-free) conditions [28]. In order to obtain connectivity values, a lagged linear coherence algorithm is applied as a measure of functional connectivity [29,30]. Moving from the scalp-recorded EEG potentials distribution, the cortical 3D mapping of current density (source localization) is carried out via eLORETA, as detailed in previous studies, also providing the proof of its exact zero-error localization property (see [30,91]). Several recent publications from independent groups [91,92,93,94,95,96,97,98,99] have supported the idea of a correct source localization using eLORETA; such an idea maintains true not only with high-density EEG recordings but also with the standard 20-channel EEG montage (10–20 system).

**Figure 1:**
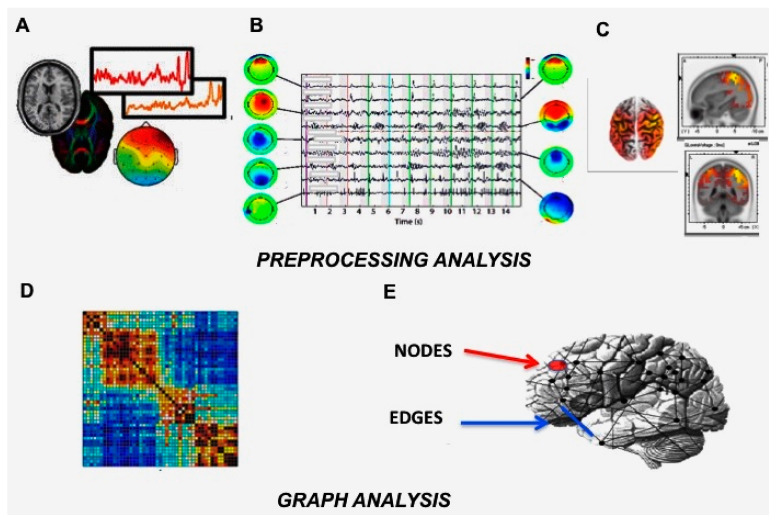
Functional connectivity data analysis: from EEG recordings (**A**), signals are preprocessed with ICA for the artifacts’ rejection (**B**), and the eLORETA algorithm is applied to extract EEG sources’ localization (**C**). Then, the graph analysis is applied with the construction of an adjacency matrix, square arrays of numbers where rows and columns correspond to nodes, and individual entries correspond to all possible connections (**D**). Nodes can correspond to specific regions, superficial signal recording sites, EEG sources, whereas edges can represent values of functional coupling between nodes (**E**).

Human activity from movement to cognitive functions is sustained by time-orchestrated coordination of neuronal aggregates simultaneously firing at multiple brain sites within distributed neuronal networks [85,100,101,102,103,104]. EEG/magnetoencephalographic (MEG) recordings allow for non-invasive measurement of the cyclic firing of neuronal assemblies with high temporal resolution (milliseconds), but with a relatively low spatial resolution (centimeters) and mainly reflect the activity of cortical neurons with little or no contribution from deep brain sources (either in the depth of sulci or in the fronto–orbital and temporo–mesial areas, including the hippocampal formation and insula). Excellent spatial resolution is peculiar of functional MRI (fMRI), reflecting fluctuations of local blood flow and metabolism through the detection of blood-oxygenation-level-dependent (BOLD) changes in the depth of the brain structure. Meanwhile, fMRI has a poor temporal resolution due to the physical properties of hemoglobin relaxation, which is reflected in a remarkable delay between the synchronized and relatively sharp neuronal firing producing the BOLD signal with a smoothing effect on the firing sharpness during the rise/decay phases of the neurovascular coupling. It is also worth mentioning that the BOLD signal is due to transient modifications of energy consumption of neuronal firing, and, therefore, it does not reflect those interneuronal connectivity mechanisms like synchronization/coherence and phase locking-unlocking, which do not require changes of firing frequency/intensity and do not imply energetic fluctuations. Coherence (Coh) [102], partial coherence (pCoh), phase-locking value (PLV) [105], mutual information (MI) [106], and directed transfer function (DTF) [104,107] include mathematical approaches to interneuronal connectivity as probed via EEG/MEG recordings. An adjunctive method is dynamic causal modeling (DCM, [108]), where the modulation of interactions in preselected networks is analyzed [109]. Inverse methods such as BEANFORM in MEG and LORETA in EEG data claim to detect deep sources, but there is the possibility that information from deep structures in the higher frequency rhythms is lost; with such methods, a good source reconstruction can be reached within the framework of their theoretical limitations (Figure 1).

In order to describe properties of large (e.g., whole-brain) networks, the original empirical data can be represented in the form of a graph. Graph theory has been widely applied to MRI tractography (for a review, see [110]), but here is mainly described for applications in EEG/MEG signal analysis. Graphs can be weighted or unweighted, and can be directed or undirected. The first step is to decide what can be considered as a node, and what can be defined as a link [42,89]. Core measures of graph theory can be computed with http://www.brain-connectivity-toolbox.net and adapted by Matlab scripts [97,111,112]. In such scripts, segregation refers to the degree to which network elements form separate clusters and correspond to clustering coefficient (C) [113]:

$$C=\frac{1}{n}\underset{i\subset N}{\sum }C_{i}=\frac{1}{n}\underset{i\subset N}{\sum }\frac{2t_{i}}{k_{i}\left(k_{i}-1\right)}$$

While integration refers to the capacity of the network to become interconnected and exchange information [114], it is defined by the characteristic path length (L) coefficient [113]:

$$L=\frac{1}{n}\underset{i\subset N}{\sum }L_{i}=\frac{1}{n}\underset{i\subset N}{\sum }\frac{\sum_{j\subset N,j\neq i}d_{ij}}{n-1}$$

The mean clustering coefficient is computed for all nodes of the graph and then averaged. It is a measure for the tendency of network elements to form local clusters [115]. Starting with the definition of L, the weighted characteristic path length Lw represents the shortest weighted path length between two nodes [113,116]. The small-worldness (SW) parameter is defined as the ratio between normalized C and L − Cw and Lw − with respect to the frequency bands. For example, to obtain individual normalized measures, one can divide the characteristic path length and the clustering coefficient by the mean from average values of each parameter in all EEG frequency bands. In this case, it should be stressed that a normalization of the data, with respect to surrogate networks, cannot be done due to the weighted values of the considered networks. The SW coefficient describes the balance between local connectedness and global integration of a network. SW organization is intermediate between that of random networks, in which the short overall path length is associated with a low level of local clustering, and that of regular networks or lattices, with a high level of clustering characterized by a long path length [96]. In this scenario, nodes are linked through relatively few intermediate steps, and most nodes maintain few direct connections. Surrogate analysis plays a pivotal role in testing the significance of functional connections in both bivariate and multi-variate estimators; it also represents a useful approach when applying a data-driven topological filter on statistically significant functional connections [117].

Generally speaking, most of the studies on brain connectivity with various techniques do not report on inter- and/or intra-subject test–retest variability; this is a significant gap for an extensive clinical application. In order to evaluate the within-subject test–retest variability [98], statistical analysis was performed on the normalized characteristic path length of EEG cortical sources for a 10-subject group with two recording sessions at a 2-week interval, introducing the factor Time (First and Second recording sessions). The statistical analyses showed no significant interaction, including time, highlighting the stability of the “Small World” analysis of EEG signals. More recently, findings from 3 recording sessions have been compared from 34 healthy subjects (mean age of 45 years) at a one-week inter-session interval [118] A between-factors analysis of variance (ANOVA) was carried out: Frequency Band (delta, theta, alpha 1, alpha 2, beta 1, beta 2, and gamma) and Time (first, second and third recording) for the Small World parameter. The statistical analysis showed that the interaction, including Time, was not significant (F(12, 396) = 0.48995, *p* = 0.92057), highlighting the stability of the proposed parameters at least when carried out in clinically stable subjects. Recently, the importance of reliability studies based on repeat-scan sessions protocol of *connectomics* in any modality has been recognized with publication of a number of freely available papers and datasets [119,120,121].

**Figure 2:**
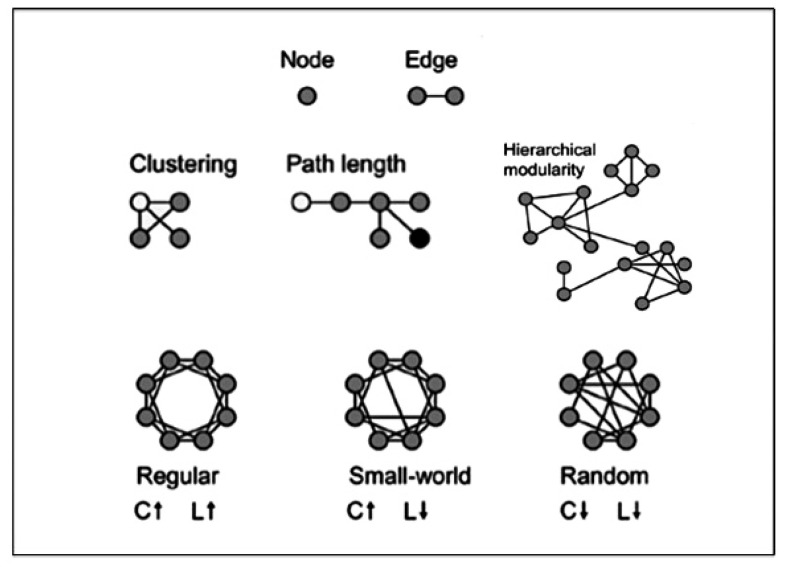
Core measures of graph theory: nodes and edges, clustering coefficient, characteristic path length, Small World index, modularity.

Transitivity (Tw) is another graph parameter: it is measured as the fraction of the node’s neighbors that are also neighbors of each other [122] and reflects, on average, the prevalence of clustered connectivity around individual nodes, a measure of segregation based on the number of triangles in the network. Tw represents a variant of the clustering coefficient not affected by individual node normalization [123]. More sophisticated methods describing segregation besides the presence of densely interconnected groups of regions also reflect their composition named the network’s modular structure (community structure). It reflects the decomposition of networks into groups of nodes, with the maximal content of within-group links (within network connections are dense), and the minimal level of between-group links (between network connections are sparse). The degree to which the network may be subdivided into such clearly delineated and non-overlapping groups is quantified by a single statistic, the modularity (Qw) (Figure 2). Unlike most other network measures, the optimal modular structure for a given network is typically estimated with optimization algorithms. Finally, local efficiency (E_loc^w) is an index of the information transfer efficiency limited to neighboring nodes (i.e., nodes with direct edges to the node of interest) and indicates how mutually interlinked neighboring nodes are [124].

Studies on network hierarchical architecture, as obtained by the analysis of simultaneous EEG oscillations of different frequencies and cross-frequency couplings during a given task performance, have opened new research avenues into cognitive mechanisms [85]. In fact, time modulation of the connectivity pattern of the nodes in a task-related network explains most of the performance variability—i.e., from “excellent” to “poor”—in apparently stable conditions [96,112,125]. In other words, the task–performance level and the task-related choice/behavior contents are largely written in the immediate architecture of the EEG networks’ connectivity, preceding the task (by a few seconds, usually).

Each EEG rhythm reflects different mechanisms and a complete view—in time, space, and frequency domains—is needed to obtain a comprehensive analysis of its functional dynamics. It is worth mentioning that, depending upon the frequency content of the examined rhythm, the time discrimination of the activation within the network frame can be as short as few milliseconds (down to 10 msec in the high γ band). Because of this, EEG connectivity analysis facilitates an evaluation of the time hierarchy governing the serial/parallel activation of the nodes and their time/space relationship within a given task-related network (i.e., whether A is active before, after, or in parallel to B).

Aging processes significantly modulate the network configuration of brain connectivity and also affect the time-varying synchronization of rhythmic oscillations in a network organization. Along this line of research, 170 healthy elderly volunteers were submitted to EEG recordings in order to define age-related normative limits [126]. Graph theory functions were applied to eLORETA on cortical sources in order to evaluate the Small World parameter as a representative model of network architecture. The analyses were carried out in the whole brain—as well as for the left and the right hemisphere separately—and in three specific resting=state sub-networks defined as follows: attentional network (AN); frontal network (FN); default mode network (DMN). To evaluate the stability of the investigated graph parameters, a subgroup of 32 subjects underwent three separate EEG recording sessions in identical environmental conditions after a few days interval. Results showed that the whole right/left hemispheric evaluation did not present side differences, but when individual sub-networks were considered, AN and DMN presented in general higher SW in low (delta and/or theta) and high (gamma) frequency bands in the left hemisphere, while for FN the alpha 1 band was lower in the left, with respect to the right hemisphere. It was also evident the test–retest reliability and reproducibility of the present methodology when carried out in clinically stable subjects.

On clinical grounds, it is of interest to the study of conditions that are considered to be prodromal to dementia as in MCI. As previously said, dementias—particularly in their early stages—mainly affect synaptic transmission and therefore represent “disconnection syndromes” [31,44,48,97,127]. A statistically significant difference in the SW organization of those MCI subjects who will progress to AD (Converted MCI, particularly those who can be defined as rapid—i.e., 1–2 years—converters) was recently found [128], the Converted MCI subjects having SW characteristics very similar to those encountered in Alzheimer’s patients 1 to 2 years before their conversion (Time 0 of the study). An abnormal increase in graph parameters in Converted, with respect to Stable MCI, for the α rhythm has been observed, along with a decrease for the δ and γ rhythms. Such findings might be interpreted in light of the background physiology of α rhythm, which is usually defined as the “idling rhythms” of the adult brain [129]. Along this vein, it is worth mentioning that, in a population of 145 MCI subjects followed up for 2 years, the receiver operating characteristic (ROC) curve derived from graph-theory EEG analysis showed SW characteristics with a >60% sensitivity (area under the ROC curve (AUC) 0.64, indicating moderate classification accuracy) for classifying the MCI state as a prodromal of AD. These findings are in line with previous studies [97,112,115] in which SW characteristics were decreased in low-frequency bands in patients with AD compared to MCI [128]. That is, the MCI connectivity pattern was less random than that of the AD group. Moreover, significant differences between healthy elderly, MCI subjects and AD patients have been demonstrated by showing that physiological brain aging presents greater specialization (though lower values) of SW characteristics that are higher than normal in low EEG frequencies and lower in α bands. Finally, converted aMCI presented a graph theory pattern practically identical to the AD one. The ROC curves gathered by a combined phenotype and genotype characteristics analysis (obtained at a low cost with widely available apolipoprotein E (ApoE) technology), produced an increase of accuracy up to 91.78% (AUC 0.97, indicating a nearly optimal classification accuracy) for identifying the MCI prodromal-to-AD state [128]. This result is in line with the fact that the ε4 allele of the APOE gene is the major risk genetic factor for the pathogenesis of late-onset AD [32,130].

This bulk of findings suggests that EEG connectivity analysis, combined with neuropsychological and genetic (i.e., ApoE alleles) evaluation, could be of great help in early MCI prodromal-to-AD identification as a first-line screening method and in intercepting those subjects with a high risk for rapid progression to AD [83,131]. How does the “graph-theoretical” model compete with other types of EEG analysis methods, and how does it contribute to AD diagnosis? Vecchio et al. [128] made a comparative analysis by applying to the same EEG epochs utilized for graph valuation methods of EEG analysis currently used for AD studies, namely, spectral coherence and power spectrum; such methods performed less than graph and showed 51.79% sensitivity, 100% specificity, and 68.86% accuracy.

Several studies converge on the idea that α rhythm is a deterministic chaotic signal involved in several functions, besides others [42], ranging from memory formation to sensorimotor processing and integration [132]. Indeed, there is evidence in the healthy, showing a positive correlation between α frequency and the speed of information processing, as well as cognitive performance [87]. In the adult EEG during resting, awake conditions α rhythms are widely recordable and dominate in the posterior brain areas, while δ rhythms are poorly represented, thus reflecting a condition of likely α-δ “reciprocal inhibition” [31]. Furthermore, it is well known that the anatomical or functional disconnection of lesioned cortical areas generates spontaneous slow oscillations in the δ range in virtually all recorded neurons. In particular, the SW decrease in the δ band represents an increase of functional inhibition. The opposite holds true for the α band.

Gamma rhythms are involved in a variety of cognitive functions, including visual object processing, attention, and memory [133], and are strictly reflecting behavioral performance (accuracy and reaction time) in several memory tasks, including episodic memory, encoding, and retrieval [134]. Gamma oscillations are pivotal in synchronization of the action potentials spike phase, a mechanism that is at the base of EEG connectivity [135]. An SW decrease in the γ band in the MCI-prodromal-to-dementia is in line with previous evidence [98], showing a decrease of SW γ band in AD with respect to MCI and control subjects. The γ band (>30 Hz) mediates information transfer between cortical and hippocampal structures for memory formation [136], particularly through feed-forward mechanisms [137] and coherent phase-coupling between oscillations recorded simultaneously from different neuronal structures [138].

We also explored [131] the EEG functional connectivity in amnesic multidomain-MCI subjects in order to characterize the DMN in converted MCI (cMCI)—those in a prodromal-to-dementia condition who converted to AD during the follow-up—compared to stable MCI (sMCI) subjects. A total of 59 MCI subjects were recruited and divided, after appropriate follow-up, into cMCI or sMCI. They were further divided into MCI with linguistic domain (LD) impairment and in MCI with executive domain (ED) impairment. The Small World (SW) index was computed, restricting to nodes of DMN regions for all frequency bands, and evaluated how they differ between MCI subgroups as assessed through clinical and neuropsychological 4-year follow-ups. Results showed that the SW index significantly decreased in γ band in cMCI compared to sMCI. In cMCI with LD impairment, the SW index significantly decreased in the delta band, while in cMCI with ED impairment, the SW index decreased in delta and γ bands and increased in the alpha1 band. It is argued that the DMN functional alterations in cognitive impairment could reflect an abnormal flow of brain information processing during resting state possibly associated with a status of pre-dementia.

The combination of all the above-mentioned feature extraction techniques results in a wide-ranging collection of features. For this reason, a feature selection process should be preferably carried out in an automated or at least in a semi-automated way. A large number of machine learning algorithms can be used to accomplish this task. A widely used procedure for both feature selection and classification in diagnosing AD applications is the support vector machine (SVM), which achieved up to 98% accuracy in early AD detection [139,140,141]. One of the major advantages of SVM is that when combined to L1-norm as penalization, it leads to sparse weight vectors and allows feature selection and classification to be accomplished in the same step [142]. An interesting variation of SVM is the Relevance Vector Machine (RVM), which replaces the binary SVM classifier with a soft-decision method based on a probabilistic Bayesian learning framework and outperformed SVM when tested in a fully-automated AD diagnostic system [139].

Recently, we investigated [143] the possibility to automatically classify physiological vs. pathological aging from cortical sources’ connectivity based on a support vector machine (SVM) applied to EEG Small World. A total of 295 subjects were recruited: 120 healthy volunteers and 175 AD. Graph theory functions were applied to the undirected and weighted networks obtained by the lagged linear coherence evaluated by eLORETA. A machine-learning classifier (SVM) was then applied. The ROC curve showed an AUC of 0.9 (indicating very high classification accuracy). The resulting classifier showed 83% sensitivity, 100% specificity, and 96% accuracy for the classification of the AD respect to control subjects. Graph theory analysis of brain connectivity from EEG signals provides useful information in distinguishing physiological and pathological age-related brain processes.

In conclusion, EEG connectivity analysis via a combination of source/connectivity biomarkers could represent a promising tool in the identification of AD patient and MCI prodromal-to-dementia subjects. This approach represents a low-cost and non-invasive method reaching high sensitivity/specificity and optimal classification accuracy, which might be combined with other biomarkers with the same characteristics (i.e., ApoE genotyping) for screening large population samples in order to obtain a risk evaluation on an individual basis.

### 2.3. TMS-EEG Co-Registration for Testing Brain Connectivity

Transcranial magnetic stimulation (TMS) is a non-invasive and painless technique introduced in 1985 by Anthony Barker, that is able to study the excitability, connectivity, and plasticity of the human cerebral cortex. If the coil for the stimulation is precisely localized on the scalp region overlaying the motor cortex, a muscle twitch in the contralateral body segment can be elicited with supra-threshold stimuli. Such responses are called motor-evoked potentials (MEPs); they can be recorded from a target muscle (i.e., the hand) by surface electromyography (EMG) and reflects the activation of corticospinal cells in the primary motor cortex (M1) by single-pulse transcranial magnetic stimulation (spTMS) [144].

The combination of TMS with EEG is considered an important tool to reveal the effective connectivity of brain networks, defined as the influence one neuronal assembly exerts over separate (eventually remote) one(s) through causal or non-causal effects. In fact, the co-registration of the EEG activity—which has a temporal resolution of a few milliseconds and can be simultaneously sampled from a large number of scalp sites—during TMS provides the opportunity of tracking temporal dynamics and inner hierarchies of brain networks that is properly their effective connectivity (for a review see Rossini et al. (2019) [83]).

TMS–EEG has several advantages: (1) Its high temporal resolution conveys precise information about the temporal order of activations of connected cortical areas (either adjacent or remote), defining at the same time the causal interactions (excitatory or inhibitory) between two areas within functional brain networks. (2) Its high temporal resolution allows the identification of critical periods during which the stimulated area and its connections to other brain regions make a critical contribution to the experimental task, thereby enabling to differentiate the connectivity pattern of different cognitive processes related to specific tasks or different brain states and whether or how they are modified by learning and training. Taking into account these points, TMS–EEG co-registration allows the evaluation of the spatio–temporal pattern of neural activity that determines the connections across brain areas, hence providing measures of effective connectivity able to test the predictions of graph theory models [145].

From the first attempt to measure TMS-evoked brain responses made in 1989 by Cracco et al. [146], several efforts have been made to overtake the severe technical limitations related to the coupling of a stimulation artifact (thousands of times higher than the signal of interest) to the recording system. Using a sample-and-hold circuit able to block the acquisition of EEG signal for several milliseconds immediately adjacent to the TMS pulse, TMS-evoked brain EEG responses were successfully measured in 1997, succeeding in tracking TMS-evoked brain activity with a temporal window of a few milliseconds after the stimulus [147,148]. Subsequent studies have begun to describe the scalp topography and to study the possible generator sources of the TMS-evoked EEG potentials (TEPs). Probably, most of the EEG signals record a linear projection of the postsynaptic currents indirectly induced by TMS; then, EEG signals can be used to locate and quantify these synaptic current distributions and evaluate local excitability and functional connectivity in the nervous system. Within the so-called “inductive approach”, applying a single TMS pulse on the brain cortex, a network of neuronal connections is triggered and the TMS-induced activation—a summation of post-synaptic potentials—spreads from the stimulation site to other interconnected parts of the brain, producing deflections in scalp EEG signals, starting a few milliseconds after the stimulus and lasting about 300 msec, first in the form of rapid oscillations and then as lower-frequency waves. Increased EEG activity following the magnetic stimulus can be observed in a number of neighboring electrodes, suggesting the spread of TMS-evoked activity to anatomically interconnected cortical areas. Particularly, TMS-evoked activity spreads from the stimulation site ipsilaterally via association fibers, contralaterally via transcallosal fibers, and to subcortical structures and spinal cord via projection fibers.

**Figure 3:**
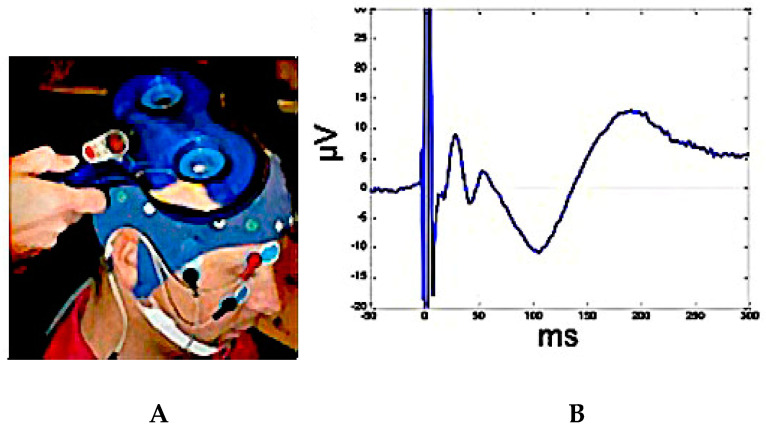
Panel (**A**): Transcranial magnetic stimulation (TMS)- electroencephalographic (EEG) co-registration. Panel (**B**): TMS–EEG evoked responses (TEPs) recorded at vertex during supra-threshold single-pulse stimulation of M1.

Therefore, TMS–EEG gives the possibility to study cortico–cortical interactions and how the activity in one area affects the ongoing activity in other areas. It has been suggested that the first part of the TMS-evoked EEG signals reflects the excitability (i.e., the functional state) of the stimulated cortex, whereas the following spatiotemporal distribution over the scalp corresponds to the spread of activation to other cortical areas, i.e., the effective connectivity of the stimulated area (for a review, see Ferreri and Rossini (2013) [148]). The amplitude, latency, and scalp topography of single-pulse TMS-evoked EEG responses have been clearly described [125,149,150]. TMS-evoked EEG averaged responses are generally highly reproducible, provided that the delivery and targeting of TMS (i.e., via neuronavigated stimulation) is well controlled and stable from pulse to pulse and between experiments. Several components of the EEG response to single-pulse TMS applied on the motor cortex have been identified and—benefiting from the knowledge of the anatomical connectivity of the brain as seen by diffusion tensor imaging studies—their spatiotemporal spread has been followed: particularly, single-pulse TMS is able to evoke EEG activity composed at the vertex by a sequence of deflections of negative polarity peaking at approximately 7, 18, 44, 100, and 280 msec, alternating with positive polarity peaks at approximately 13, 30, 60, and 190 msec post-TMS (Figure 3) [150].

However, the previously described pattern of TEPs is not invariable because, in addition to inter-individual differences, it depends on the stimulation intensity, the exact coil location and orientation [149], the local and general state of the cortex [151], the level of vigilance [152], as well as the age of the stimulated brain [153]. Given these unique features, TMS–EEG appears very suitable to test and evaluate the functional brain architecture suggested by graph theory models, both at rest and during cognitive processes. Several TMS–EEG studies on the motor system at rest have demonstrated that the TMS-induced activity spreads from the stimulated node to other nodes of the same motor network: the TMS of the primary motor cortex causes the successive activation of ipsilateral supplementary/premotor areas and contralateral motor region with a short conduction time. The nature of these connections seems to be inhibitory rather than excitatory and depends on the level of cortical activation immediately before and during the stimulation [125,153]. As therefore observed, the restriction of the TMS-activity to the specialized motor network suggests a modular node organization of functional brain architecture at rest. Other studies on the motor cortex at rest have revealed that the stimulation of M1 also generates the late activation of areas outside the “motor” network, including the cingulate gyrus and the temporo–parietal junction. The spread of the later components of TEPs to other areas over the motor network suggests the involvement of further nodes and brain hubs implicated in the transmission of information across the brain. Additional evidence about this bottom-up signal propagation from lower-degree nodes to brain hubs has been provided by studies on the visual system. On the contrary, the existence of mechanisms of top-down modulation has been shown in several studies stimulating multimodal associative areas responsible for high cognitive processes during task performance: it has been demonstrated that the diffusion of TMS-induced activity from these areas across the brain could be divergent depending on the task context, preferring the engagement of one network rather than another. This kind of TMS–EEG approach defines the “interactive approach” and seems to confirm that the targeting of associative areas could correspond to the brain hubs, a subset of high-degree brain sites able to mediate communication between multiple modules or networks according to the cognitive context. These findings, taken together, highlight the potential role of TMS–EEG to test the dynamic changes of cortico–cortical connectivity according to graph theory predictions, identifying both a specialized modular/network segregation during the resting state and a modular/network integration during high cognitive demands with dense connectivity through brain hubs activation.

In addition to standard TEPs, single-pulse TMS or frequency-tuned train of pulses can also trigger or enhance brain oscillatory activity or perturb ongoing rhythms of the targeted cortical area, eliciting event-related phenomena, such as EEG rhythm synchronization or desynchronization. Brain oscillations represent a mechanism through which the communication of neuronal assemblies—by synchronization in specific frequency bands—is rendered more effective, precise, and selectively tuned on the transmission of the relevant information. It has already been demonstrated that different cortical areas, when stimulated, respond at a characteristic frequency, i.e., their natural frequency, and that functionally segregated networks could oscillate at different frequencies at rest [154]. Given these assumptions, some authors speculated that TMS could interact with such oscillatory patterns in the directly stimulated cortical area and in distant areas belonging to the same neural network thus inducing a resonant frequency activity in all “synchronized” areas of the same network by mechanisms of longer-range synchronization (interregional coherence). This frequency-specific “resonant effect” should ensure better information transfer across brain structures and could even determine changes in the behavioral performance [155]. Therefore, the “rhythmic TMS–EEG approach” appears as a promising tool in mapping the natural frequency of different cortical areas and identifying the role of a specific frequency oscillatory activity in distinct brain functions.

With all these premises, despite some technical limitations, it is easy to realize how TMS–EEG can be used to examine normal and altered effective brain connectivity under both physiological and pathological specific conditions, indicating the strengthening or weakening of existing cortico–cortical connections or the recruitment of compensatory networks. Indeed, besides assessment of the general state of the brain, TMS–EEG can be used to track the interactions of brain areas during sensory processing, cognition, or motor control and, moreover, to evaluate such neurological disorders as Alzheimer’s disease (AD), characterized by altered connectivity. The few studies regarding this field [156,157,158,159,160], integrating previous observations obtained with the use of the TMS alone (for example, [161,162], already showed that the cortical stimulation in AD patients was associated with significant disruption in TMS-induced activity over several brain areas compared with healthy controls, suggesting a potential role of TMS–EEG as a neurophysiological marker for diagnosis and early identification of mild cognitive impairment (MCI) and AD (Table A1 in Appendix A).

In this context, our research group was able to describe—for the first-time—specific neurophysiological hallmarks of motor cortex functionality in early AD [158]. By using TMS–EEG co-registration, we have demonstrated that in mild AD patients without motor symptoms, the sensorimotor system is strongly hyperexcitable and deeply rearranged with the recruitment of additional neural sources, the activation of reverberant local circuits, and their integration in the distributed network subtending sensorimotor functions. Thus, we have proposed this plastic cortical reorganization would be ensured by the particular organization of the sensorimotor system based on a distributed network with a replicated topographic organization of the same body part and could be interpreted as a compensatory mechanism allowing for the preservation of sensorimotor programming and execution since the preclinical stage trough the MCI stage and over a long period of time in spite of disease progression [158]. Because of such encouraging findings, we are now employing TMS–EEG to investigate hallmarks of sensorimotor cortex functionality in aMCI, assuming they represent the subtending long-term plastic rearrangement induced by the neurodegeneration during the pauci symptomatic prodromal stage and can thus affordably predict the future conversion to AD. TMS–EEG recordings and analysis will be performed both to describe the excitability and effective connectivity of the somatosensory network of the whole aMCI group with respect to a control group, and to investigate baseline differences in these neurophysiological properties between the two groups. Particularly we want to determine (1) whether the sensorimotor networks would show peculiar alterations in aMCI as a whole group, and (2) if there is any hallmark of sensorimotor network disruption able to predict long-term disease progression at the individual level. We are now finalizing a five-year clinical follow-up in a restricted group of aMCI, and, effectively, our preliminary results are promising and indicate that some parameters of the M1 functionality can be used as reliable biomarkers of AD.

## 3. Conclusions

The time is now right for searching for instrumental biomarkers for early—hopefully, preclinical—diagnosis of dementia in order to contrast as soon as possible all the modifiable risk factors for neurodegeneration, as well as to initiate (as soon as they will become available) disease-modifying drugs. To reach this goal, all the health systems are actually looking for a combination of biomarkers having clear characteristics: high accuracy/specificity/sensitivity, affordable costs, non-invasiveness, and large territorial availability. Neurophysiological techniques have all the required characteristics and are optimal candidates, at least for a 1st level screening.

## Appendix A

**Table A1:** Summary of the main studies that analyzed neurophysiological changes associated with the development of dementia. List of abbreviations: electroencephalography (EEG), fluorodeoxyglucose positron emission tomography (FDG–PET), mild cognitive impairment (MCI), Alzheimer’s disease (AD), normal elderly (Nold), Small Worldness (SW), transcranial magnetic stimulation (TMS), repetitive transcranial magnetic stimulation (rTMS), motor cortex (M1), Mini Mental State Examination (MMSE), default mode network (DMN).

Author (Year)	Methods	Biomarkers	Subjects	Main Findings	Reference
Babiloni C (2016)	EEG, FDG-PET	spectral analysis (power)	AD	19 AD patients were compared with a group of 40 Nold. The AD group performed FDG-PET. In the AD patients, there was a positive correlation between the Alzheimer’s discrimination analysis tool (PALZ) score and the activity of delta sources in the cortical region of interest (*p* < 0.05) suggesting a relationship between resting-state cortical hypometabolism and synchronization of cortical neurons at delta rhythms in AD patients with dementia.	[22]
Rossini PM (2006)	EEG	spectral analysis (linear coupling)	AD, MCI	In 69 MCI, baseline fronto-parietal midline coherence, delta (temporal), theta (parietal, occipital and temporal), and alpha 1 (central, parietal, occipital, temporal, limbic) sources were stronger in MCI Converted than stable subjects (*p* < 0.05). Low midline coherence and weak temporal source were associated with a 10% annual rate AD conversion, while this rate increased up to 40% and 60% when strong temporal delta source and high midline gamma coherence were observed respectively.	[31]
Jelic V (2000)	EEG	spectral analysis (power)	MCI	In 27 MCI patients, progression to AD in a follow up of 21 months was associated with a significantly higher theta relative power and lower beta relative power and mean frequency at the temporal and temporo - occipital derivations	[34]
Adler G (2003)	EEG	spectral analysis (power)	AD	A study with 31 AD compared with 17 Nold. AD patients showed a widespread increase in delta and theta power density and posterior decrease in α and β power density with a lowering of α power density peak.	[35]
Stam CJ (2007)	EEG	spectral analysis (graph theory)	AD	In a study with 15 AD vs. 13 Nold, the characteristic path length L was significantly longer in the AD patients, whereas the cluster coefficient C showed no significant changes. This pattern was still present when L and C were computed as a function of K. A longer path length with a relatively preserved cluster coefficient suggests a loss of complexity and a less optimal organization.	[42]
Jelles B (1999)	EEG	spectral analysis (power)	AD	In a study with 24 probable AD vs. 22 Nold, the correlation dimension (D2) was significantly lower in the Alzheimer patients compared to controls	[43]
Dauwels J (2010)	EEG	spectral analysis (nonlinear coupling)	MCI	Two synchrony measures, Granger casuality, and stochastic event synchrony are able to distinguish MCI patients from age-matched control subjects.	[44]
Azami H (2016)	MEG	entropy	AD	In 36 AD vs. 26 Nold, multiscale dispersion entropy (MDE) values in AD compared with multiscale permutation entropy (MPE) and multiscale entropy (MSE) was significantly lower than their corresponding MSE- and MPE-based values.	[45]
Babiloni C (2009)	EEG, MRI	spectral analysis (power)	AD, MCI	In a study with 35 AD, 80 MCI and 60 Nold, the EEG sources showed a significant linear correlation with hippocampal volume also supported a non-linear correlation with hippocampal volume strongly for the logarithmic one, suggesting that progressive atrophy of hippocampus correlates with decreased cortical alpha power, as estimated by using LORETA source modeling, in the continuum, along MCI and AD conditions.	[47]
Babiloni C (2009)	EEG, MRI	spectral analysis (power)	MCI	Study with 54 MCI subjects with follow-up of 1-year vs. 45 Nold and 50 AD. In MCI, the EEG recordings showed a decreased power of posterior alpha1 and alpha2 sources, suggesting that the resting state EEG alpha sources were sensitive-at least at the group level-to the cognitive decline occurring in the amnesic MCI group over 1 year.	[51]
Jeong J (2004)	EEG	spectral analysis (nonlinear)	AD	EEG in AD showed a lower correlation dimension (D2) and the largest Lyapunov exponent (L1) values than in the healthy. Despite their different focus on static and dynamic properties of the EEGs, the results of both D2 and L1 were associated with a reduction of complexity in EEG activity due to AD	[54]
Smits FM (2016)	EEG	fractal dimension	AD	A comparision between 67 AD vs. 41 Nold showed a reduced fractal dimension in AD compared to healthy especially in temporal-occipital regions	[61]
Escudero J (2006)	EEG	entropy	AD, MCI	In a study with 11 AD and 11 Nold, entropy metrics of spontaneous EEGs in AD and in MCI showed reduced irregularity in AD patients’ EEG activity	[69]
Vecchio F (2015)	EEG, MRI/DTI	spectral analysis (graph theory)	AD, MCI	40 subjects, including 9 Nold, 10 MCI, 10 mild AD, 11 moderate AD. Callosal fractional anisotropy (FA) reduction, observed in subjects with Alzheimer’s disease (AD) and mild cognitive impairment (MCI), is associated with a loss of brain interhemispheric functional connectivity characterized by increased delta and decreased alpha path length.	[97]
Vecchio F (2014)	EEG	spectral analysis (graph theory)	AD, MCI	Analysis of a database of 378 participants, including AD, MCI, and Nold. Path Length showed a different pattern between normal cognition and dementia as observed in the theta band (MCI subjects are found similar to healthy subjects), while for the normalized Clustering coefficient a significant increment was found for AD group in delta, theta, and alpha 1 bands; the small world parameter presented a significant interaction between AD and MCI groups showing a theta increase in MCI.	[98]
Miraglia F (2016)	EEG	spectral analysis (graph theory)	AD, MCI	30 Nold, 30 aMCI, and 30 AD during eyes closed EC and eyes open EO. In Nold, in EO condition, the brain network is characterized by higher SW in alpha bands and lower SW in beta2 and gamma bands. In aMCI, SW has the same trend, except for delta and theta bands where the network shows less SW. AD shows a similar trend of Nold, but with less fluctuations between EO/EC conditions. aMCI presents SW midway between AD and Nold. In delta and theta bands, in EC, the aMCI group presents network’s architecture similar to Nold, while in EO aMCI, SW similar to AD	[112]
de Hann W (2012)	MEG	spectral analysis (graph theory)	AD	In 18 AD vs. 18 Nold, graph spectral analysis confirmed the hub status of the parietal areas and demonstrated a low centrality of the left temporal region in the theta band in AD patients that was strongly related to the MMSE. In AD, impaired network synchronization and a clinically relevant left temporal centrality loss were found	[115]
Vecchio F (2017)	EEG	spectral analysis (graph theory)	AD	In 110 AD and 34 healthy Nold, Alpha band connectivity was negatively correlated, while slow (delta) and fast-frequency (beta, gamma) bands positively correlated with the hippocampal volume of Alzheimer subjects. The larger the hippocampal volume, the lower the alpha, and the higher the delta, beta, and gamma Small World characteristics of connectivity.	[126]
Vecchio F (2018)	EEG, Apo-E allele	spectral analysis (graph theory)	aMCI	145 aMCI classified as Converted to AD (C-MCI, 71) or Stable (S-MCI, 74) according to follow up. Small-World EEG analysis, in combination with an Apo-E allele testing, evaluate on an individual basis with great precision the risk of MCI progression (96.7% sensitivity, 86% specificity and 91.7% accuracy (AUC = 0.97))	[127]
Julkunen P (2011)	TMS-EEG	Cortical Excitability (P30 amplitude)	AD, MCI	In this study with 4 control subjects, 5 MCI and 5 AD, the TMS–EEG response P30 amplitude correlated with cognitive decline and showed good specificity and sensitivity in identifying healthy subjects from those with MCI or AD.	[156]
Casarotto S (2011)	TMS-EEG	Cortical Excitability	AD	In this study with 9 healthy young, 9 healthy elderly, and 9 AD, frontal cortex excitability was not significantly different between healthy young and elderly individuals while was clearly reduced in AD patients.	[157]
Ferreri F (2016)	TMS-EEG	M1 Cortical Excitability and Connectivity	AD	In this study with 12 mild AD patients, the sensorimotor system was found hyperexcitable, and its connectivity disrupted with respect of 12 healthy elderly, despite the lack of clinically evident motor manifestations.	[158]
Bagattini C (2019)	TMS-EEG	Cortical Excitability (P30 amplitude)	AD	In this study with 26 AD patients, the TMS–EEG response P30 amplitude predicted MMSE and face-name memory scores. Particularly higher P30 amplitude predicted poorer cognitive and memory performances.	[159]
Koch G (2018)	rTMS, TEM-EEG	Cortical Excitability and Connectivity	AD	In 14 early AD, a 2-week treatment with rTMS on the precuneus induced a selective improvement in episodic memory. TMS-EEG recording revealed a precuneus enhanced activity and a modification of its functional connectivity within the DMN	[160]
Ferreri F (2003)	TMS	M1 Cortical Excitability (MEP amplitude)	AD	In 16 AD, motor cortex excitability, measured with TMS, was increased, and the center of gravity of motor cortical output, as represented by excitable scalp sites, showed a frontal and medial shift, without correlated changes in the site of maximal excitability (hot-spot).	[161]
Ferreri F (2011)	TMS	M1 Cortical Excitability (MEP amplitude)	AD	In 10 AD patients before and after long-term AchEIs therapy, M1 excitability was found to be unchanged in patients with stabilized cognitive performance during the therapy.	[162]
